# Phase 1 cardiovascular rehabilitation: be aggressive?

**DOI:** 10.1186/1749-8090-6-140

**Published:** 2011-10-17

**Authors:** Vitor Oliveira Carvalho

**Affiliations:** 1 Laboratório de Insuficiência Cardíaca e Transplante do Instituto do Coração do Hospital das Clínicas da Faculdade de Medicina da USP (InCor HC-FMUSP), São Paulo, Brazil

## Background

It is well known that the most common respiratory complications after cardiac surgery are related to sternotomy, extracorporeal circulation and its inflammatory reaction [1]. Phase 1 cardiovascular rehabilitation is widely indicated to minimize the adverse effects of cardiac surgery, including respiratory function [2,3]. However, is there a critical day to physiotherapists?

The study by Moreno et al [4] is very important and adds important information to what we know about respiratory function and physiotherapy after cardiac surgery. This study aimed to assess the pulmonary function in patients after coronary artery bypass graft surgery treated with a physiotherapy protocol. The authors showed that the day 3 after cardiac surgery showed the worst values of forced vital capacity, maximal inspiratory pressure and maximal expiratory pressure in the follow up of 30 days. However, the authors did not show data about the incidence of atelectasis and pulmonary complications along the follow up. The results showed by Moreno et al [4], raised some questions: should physiotherapists be more aggressive in the third day after surgery? If yes, could the aggressive intervention impact in lung complications and survival?

This way, new trials are important to elucidate the best physiotherapy strategy in patients after cardiac surgery. Moreover, to investigate if an aggressive physiotherapy in the worst period of lung function after cardiac surgery decreases the incidence of atelectasis, pulmonary complications and improves patients survival.
